# Sore Nipples

**Published:** 1868

**Authors:** 


### SORE NIPPLES.

Dr. Blaguiens says, in the *Journal des Connaisances Medicales*, that three or four applications of the following compound cures this complaint: Cocoa butter, 150 grains; ext. of rhatany, 10 grains.—*Nashville Journal.*

